# Male Breast Cancer: 10-Year Experience at Mansoura University Hospital in Egypt

**DOI:** 10.3969/j.issn.2095-3941.2012.01.004

**Published:** 2012-03

**Authors:** Wafaa El-Beshbeshi, Engy M Abo-Elnaga

**Affiliations:** Department of Clinical Oncology and Nuclear Medicine, Faculty of Medicine, Mansoura University, Mansoura, El-Daklalia 002, Egypt

**Keywords:** breast neoplasms, male, therapeutics, Egypt

## Abstract

**Objective:**

Male breast cancer (MBC) is a rare disease representing less than 1% of all malignancies. The objective of the study is to report clinicopathological characteristics, treatment patterns, and outcomes of MBC in Mansoura University Hospital, Egypt.

**Methods:**

This retrospective study focused on male breast cancer patients during 10 years (2000-2009). The studied variables were data regarding general characteristics of patients, treatment modalities and survival.

**Results:**

The series included 37 patients (0.8% of all breast cancer). The median age was 57.7 years (range: 26-86 years). The main clinical complaint was a mass beneath the areola in 94.5% of the cases. Most patients had a locally advanced disease. 94.5% of tumors were invasive duct carcinomas. The treatment was essentially surgery in 91.8%, followed by adjuvant radiotherapy (in 89.2%), hormonal therapy (in 56.7%) and chemotherapy (in 91.8%). Follow-up period ranged from 6-115 months. Local recurrence occurred in 4 cases and metastasis in 11 cases. The 2-year and 5-year overall survival (OS) rates were 81.6% and 60.5%, respectively. The 2-year and 5-year disease-free survival (DFS) rates were 68.4%, and 52.6%, respectively. OS was not significantly affected by any of the studied parameters. Factors influencing DFS were: T stage (*P*=0.05), positive lymph nodes (*P*=0.043), metastasis (*P*=0.004), and chemotherapy (*P*=0.046).

**Conclusions:**

MBC is a rare disease and often diagnosed at a locally advanced stage. The management of male and female breast carcinoma is identical. Future research for better understanding of this disease is needed to improve the management and prognosis of male breast cancer patients.

## Introduction

Breast cancer in men is a rare disease, accounting for <1% of all breast cancer cases in the United States ^[^^1^^]^ and approximate 0.1% of cancer mortality in men ^[^^2^^]^.

Although the epidemiologic literature on female breast cancer (FBC) is extensive, little is known about the etiology of male breast cancer (MBC). This difference is mostly due to the rarity of this disease in men. The causes of MBC remain unknown, but several risk factors exist ^[^^3^^]^. A positive family history of breast cancer is associated with increased risk of MBC. A population-based series observed that 17% of MBC patients have at least one first-degree relative with breast cancer ^[^^4^^]^. Genetics seems to play an important role in MBC; BRCA2 mutations seem to be associated with the majority of inherited breast cancer in men ^[^^5^^]^. The most frequently examined epidemiologic risk factors for MBC include disorders associated with increased estrogen levels, testicular disorders, gynecomastia ^[^^6^^]^, Klinefelter syndrome, obesity, environmental or occupational exposure, and dietary factors^[^^7^^]^.

The presentation of breast cancer in males is typically a painless and hard subareolar mass, but it can also manifest as nipple retraction or ulceration and bloody nipple discharge^[^^8^^]^. The appearance of the cutaneous signs is the most frequent reason for consultation ^[^^9^^]^.

MBC most commonly presents with invasive duct histology; lobular and ductal carcinoma *in situ* were less common than in women ^[^^10^^]^, MBC generally has a higher rate of estrogen/progesterone hormone receptor positivity than FBC, with 80%-90% expressing the estrogen receptor and 65%-92% expressing the progesterone receptor ^[^^11^^]^.

Surgery remains the gold standard treatment for MBC. Modified radical mastectomy with axillary dissection is the most common procedure performed for MBC ^[^^12^^]^. Adjuvant therapy is based on retrospective studies of MBC conducted over the past 20 years using the guidelines for breast cancer in women ^[^^13^^]^. Postmastectomy radiation should follow recommendations used for treatment of FBC ^[^^14^^]^. Postoperative radiotherapy achieves local control but no effect is observed on survival ^[^^15^^]^.

The mainstay of systemic therapy for hormone receptor–positive MBC is hormonal therapy. Tamoxifen is the most extensively studied and has been shown to be clinically effective in endocrine-responsive MBC, but may be associated with poor compliance ^[^^16^^]^. There are still no data supporting using aromatase inhibitors with or without concurrent leutinizing hormone–releasing hormone (LHRH) agonist for treatment of MBC ^[^^17^^]^.

Chemotherapy seems to benefit patients with endocrine-non-responsive disease, large tumors, and/or node-positive disease and in younger patients ^[^^18^^]^. Frequently used chemotherapy regimens were CMF, FAC, FEC and EC ^[^^19^^]^. The taxanes may be considered when lymph nodes are involved^[^^20^^]^.

In our retrospective study, we aimed to study clinic-pathological characteristics, treatment patterns, and outcomes of MBC in Mansoura University Hospital, Egypt.

## Patients and Methods

This retrospective study covered 10 years from January 2000 to December 2009. The study was carried out at the Clinical Oncology and Nuclear Medicine Department, Mansoura University Hospital, Egypt. The study included 37 patients with MBC with histological confirmation. Data were collected from the files of patients in our department. The studied variables were data regarding general characteristics of patients: age, residence, risk factors, presenting symptoms and signs, duration of symptoms, location, histopathology and grade of tumors (histological type and grading followed the World Health Organization (WHO) classification) ^[^^21^^]^, hormonal status, TNM staging (tumor stage was based on the 6th AJCC criteria) ^[^^22^^]^, treatment modalities and survival (OS and DFS).

Surgery either lumpectomy, simple mastectomy ± axillary clearance, radical or modified radical mastectomy was performed for all non-metastatic patients. The chemotherapy was used either anthracycline containing regimen as FAC, FEC or CMF. FAC (5-Flourouracil 500 mg/m^2^ i.v. day 1, Doxorubicin 50 mg/m^2^ i.v. day 1, Cyclophosphamide 500 mg/m^2^ i.v. day 1) repeated every 21 days for 6 cycles. FEC (5-Flourouracil 500 mg/m^2^ i.v. day 1, Epirubicin 50-100 mg/m^2^ i.v. day 1, Cyclophosphamide 500 mg/m^2^ i.v. day 1) repeated every 21 days for 6 cycles. CMF (Cyclophosphamide 500 mg/m^2^ i.v. day 1 and 8, Methotraxate 40 mg/m^2^ i.v. day 1 and day 8, 5-Flourouracil 600 mg/m^2^ i.v. day 1 and 8) repeated every 28 days for 6 cycles.

Adjuvant radiation therapy for chest wall and peripheral lymphatics was given in indicated cases. The dose given was 50 Gy in 25 setting over 5 weeks. The hormone therapy was used in the form of 20 mg tamoxifen daily for 5 years.

Clinical follow-up included physical examination, laboratory tests (complete blood count, renal and liver function tests) and radiological studies (including chest X-ray, abdominal ultrasound and bone scan) every 6-12 months for detection of relapse. DFS was calculated from the date of surgery till the date of recurrence (either local or distant) and OS was calculated from the date of diagnosis till the date of death or loss to follow up.

### Statistical analysis

The statistical analysis of data was done by using SPSS (SPSS Inc., Chicago, IL, USA) program statistical package for social science version 16. Chi-square test was employed for qualitative data to test proportion independence. DFS and OS were estimated and plotted by using Kaplan-Meier method and log-rank test ^[^^23^^]^. A *P*-value <0.05 was considered statistically significant.

## Results

The study included 37 male patients with a histopathological diagnosis of breast cancer. A total of 4,761 cases of breast cancer had histopathological confirmation of whom 4,723 cases were females (99.2%) and 37 cases were males (0.8%). The risk factors found are represented by history of liver disease in 6 cases (16.2%), gynaecomastia in 2 cases (5.4%), and breast trauma in 1 case (2.7%). No family history of breast cancer existed. The base line characteristics of all patients and their tumors are summarized in **Table 1**. The median age was 57.7 years (range: 26-86 years). The majority of patients came from rural areas (25 cases, 67.6%).

**Table 1:** Patients’ characteristics.

Characteristics	No. of patients (%)
Age	
Median (range)	57.7 (26-86 years)
Locality	
Rural	25 (67.6)
Urban	12 (32.4)
Tumor site	
Left breast	24 (64.9)
Right breast	13 (35.1)
Presenting symptoms	
Retroareolar lump	35 (94.5)
Nipple retraction	3 (8.1)
Bleeding per nipple	3 (8.1)
Skin redness	2 (5.4)
Gynaecomastia	2 (5.4)
Tumor histology	
Infiltrating duct carcinoma	35 (94.6)
Invasive papillary carcinoma	1 (2.7)
Undifferentiated carcinoma	1 (2.7)
Grade	
G_1_	1 (2.7)
G_2_	26 (70.3)
G_3_	10 (27.0)
Tumor size	
T_1_	1 (2.7)
T_2_	6 (16.2)
T_3_	13 (35.2)
T_4_	17 (45.9)
Axillary nodal status (N)	
N+	18 (48.7)
N-	15 (40.5)
Unknown	4 (10.8)
Metastasis (M)	
M_0_	34 (91.9)
M_1_	3 (8.1)
Hormonal status	
Unknown	16 (43.2)
Positive	18/21 (85.7)
Negative	3/21 (14.3)

The main clinical complaint was a firm retroareolar lump in 35 cases (94.5%), associated with nipple retraction, bleeding per nipple in 3 cases for each (8.1%) and skin redness, gynaecomastia in 2 cases for each (5.4%). The median duration of the evolution of these symptoms and signs was 9 months. The clinical examination revealed palpable lymph nodes in the axillary region in 15 cases (40.5%). The tumor was found on the left breast in 24 cases (64.9%) and on the right breast in 13 cases (35.1%); there was no case of bilateral breast cancer.

The most common pathological type was infiltrating duct carcinoma (IDC) in 94.6%, invasive papillary and undifferentiated carcinoma in 1 case for each (2.7%). The majority of cases were classified as T_3_ (35.2%) and T_4_ (45.9%). Forty-eight percent had lymph node metastasis at time of diagnosis. The hormonal receptor was carried out in 21 patients (56.8%); with 18 cases positive (85.7%) and 3 cases negative (14.3%). Investigations to assess the extent of the disease showed 3 cases (8.1%) of synchronous bone metastases at the time of diagnosis.

As regards treatment, different treatment modalities are summarized in **Table 2**. The treatment was essentially surgery which was done in 34 cases (91.8%), and modified radical mastectomy was the most frequent surgical interference (54%). After completion of surgery, adjuvant therapies were administered: 33 patients received radiotherapy (89.2%), 21 patients received hormone therapy with 20 mg of tamoxifen daily (56.7%) and 34 patients received chemotherapy (91.8%) in the form of anthracyclin based regimen FAC (fluorouracil, adriamycin, cyclophosphamide), FEC (fluorouracil, epirubicin, cyclophosphamide) in 64.9% of patients or CMF (cyclophosphamide, methotraxate, fluorouracil) in 27.0% of patients. No patients received neoadjuvant chemotherapy. Follow up period ranged from 6 to 115 months. The evolution had been characterized by local recurrence, after a median time of 23 months in 4 cases (10.8% of all patients). Metastasis occurred, after a median time of 22.5 months in 11 cases (29.7% of all patients). The site of metastasis was bone in 7 cases, lung in 2 cases, bone and lung in 2 cases.

**Table 2:** Lines of treatment.

Treatment	No. of patients (%)
Surgery	
No	3 (8.1)
Modified radical mastectomy	20 (54.0)
Radical mastectomy	8 (21.7)
Simple mastectomy	4 (10.8)
Lumpectomy	2 (5.4)
Adjuvant radiotherapy	
Yes	33 (89.2)
No	4 (10.8)
Adjuvant hormonal therapy	
Yes	21 (56.7)
No	16 (43.3)
Adjuvant chemotherapy	
No	3 (8.1)
Anthracyclin based (FAC or FEC)	24 (64.9)
CMF	10 (27.0)

**Figure 1:**
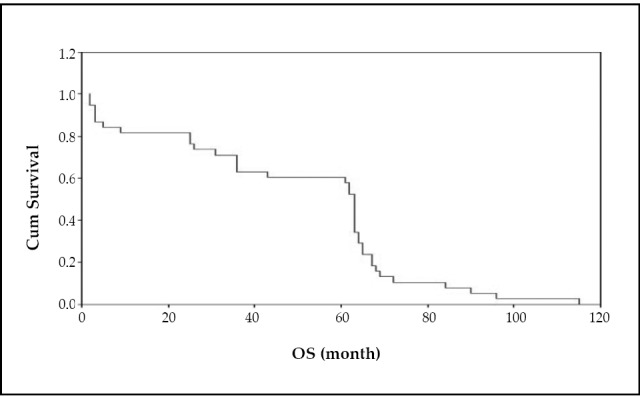
Overall survival.

**Figure 2:**
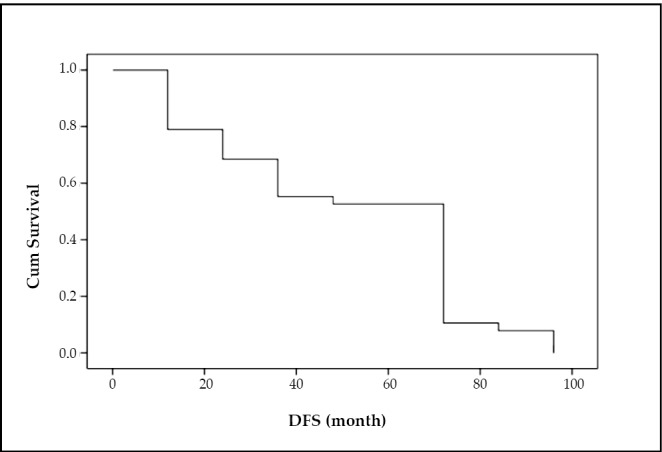
Disease free survival.

The 2- and 5-year OS rates were 81.6% and 60.5%, respectively. The median OS time was 63 months (mean: 49.9 months) (**Figure 1**). The 2- and 5-year DFS rates were 68.4%, and 52.6%, respectively. The median DFS time was 62 months (mean: 43.7 months) (**Figure 2**).

Overall survival was not significantly affected by any of the studied parameters, while factors influencing DFS were T stage (*P*=0.05), positive lymph nodes (*P*=0.043), presence of metastasis at time of presentation (*P*=0.004) and chemotherapy administration (*P*=0.046).

## Discussion

The incidence of MBC varies widely among countries which accounts for 1.2% of newly diagnosed BC in the United States and 1% in Europe ^[^^24^^]^. However, MBC incidence is much higher in sub-Saharan Africa, approximately 5%-15% ^[^^9^^]^. This high incidence is attributed to possible hyperestrogenism as a result of liver damage from endemic infectious disease in these areas as bilharziasis and viral hepatitis ^[^^5^^]^. In the opposite side, MBC incidence in Asia is lower (0.38%-0.60%). The incidence of MBC may be lower in Asia than in the West due to biological and/or environmental factors, since the incidence of FBC is much higher in Western countries ^[^^25^^, ^^26^^]^. In our study, the incidence of MBC was 0.8% of all breast cancers which was similar to the result obtained from the study conducted in Tunisia (1.0%) ^[^^27^^]^.

The causes of MBC remain unknown, but several risk factors exist ^[^^10^^]^. In our study, the risk factors found were history of liver disease (15.7%), gynaecomastia (5.3%), and breast trauma (2.6%). However, no family history of breast cancer existed.

The median age at diagnosis of MBC is 60 years (range: 63-68 years), which is approximately 5-10 years older than the average age at diagnosis of FBC. Males’ age distribution is unimodal while in females the distribution is bimodal with two peaks at 52 and 71 years ^[^^28^^, ^^29^^]^. The median age of our patients was 57.7 years which is similar to the report from studies in different countries in the world and North Africa^[^^27^^-^^32^^]^. It is lower in the report from study in sub-Saharan Africa (52.8 years) ^[^^9^^]^.

The majority of patients came from rural areas that are coped with results obtained by Rachid et al. ^[^^9^^]^ This may be related to the high prevalence of viral hepatitis and endemic bilharziasis in rural areas.

MBC occurs most often in the form of hard mass beneath the areola in 94.5% of our cases, this is similar to the symptom that presented in most series ^[^^9^^, ^^28^^-^^31^^]^. Because of the small breast size, mass rapidly invades the skin, so diagnosis of MBC is frequently established in more advanced stages than FBC ^[^^29^^]^. The median duration of symptoms before diagnosis was 9 months, vs. 8 months for Ben-Dhiab in Tunisia ^[^^27^^]^, 10 months for Donegan on an American series ^[^^31^^]^, and 28 months for Bourhafour in Morocco ^[^^29^^]^.

The tumor site was slightly predominant to the left breast (64.9%), which copes with Ben Dhiab et al. ^[^^27^^]^. The most frequently encountered histological type of MBC is IDC, representing about 85%-95% in several series ^[^^27^^-^^32^^]^. The other varieties are less common than in FBC, particularly the lobular type, as the male breast gland is devoid of lobules^[^^10^^]^. The result in our study showed an incidence of 94.6% for IDC, significantly higher than the other histological types, with no case of lobular carcinoma.

TNM staging found a high distribution of T_3_ and T_4_ (81.1%) in our patients, indicating a locally advanced stage of cancer. In Africa the rate varies from 54% to 100% ^[^^9^^, ^^11^^, ^^27^^]^, but it was about 40% in Western countries ^[^^15^^, ^^31^^-^^34^^]^. Several reasons can account for this delayed diagnosis in Africa including ignorance of the patients, low economic levels of the population, and error in initial diagnosis ^[^^9^^]^.

The positive node metastasis (pN+) was 48.7% among our patients who underwent axillary clearance. In the literature, the rate varies from 35% to 84% ^[^^25^^, ^^32^^-^^34^^]^. Axillary lymph node assessment is usually performed via either axillary sampling/clearance or sentinel node biopsy. Sentinel lymph node biopsy is established as an accurate and low morbid procedure for FBC, and it also plays an important role in MBC ^[^^15^^]^. However, Sentinel lymph node biopsy was not yet established for in our department until 2009.

The hormone-dependence of MBC is established and the hormone receptors are positive in 65% to 90% of cases in many series ^[^^9^^, ^^27^^-^^30^^]^. In our study, these receptors were tested in only 21 patients and 18 cases were positive. This may be attributed to the fact that the hormone receptor assessment was not done in routine investigation before the year of 2003.

The treatment guideline has been extrapolated from the data based on MBC invoving surgery, radiotherapy, chemotherapy, and hormone therapy. Currently, the modified radical mastectomy with axillary node dissection has replaced the radical mastectomy, with comparable results ^[^^13^^]^. The preservative methods applied for FBC should not be used for MBC because of small breast, central tumors, invasion of the skin, and the pectoral muscle ^[^^27^^, ^^33^^, ^^34^^]^. Modified radical mastectomy (MRM) was carried out in the majority of the cases (54%) and lumpectomy in only 2 cases. Radiotherapy was carried out in 33 cases (89.2%) in this study. Several studies have found that radiotherapy reduces the risk of local recurrence but does not change the OS ^[^^16^^, ^^18^^]^. The effect of radiotherapy on local recurrence was not detected in our study as only 4 patients did not receive radiotherapy.

Chemotherapy became a standard adjuvant treatment using several protocols and is usually decided by assessing the risks and benefits in the same manner as for FBC. Adjuvant chemotherapy with CMF or anthracycline-based is indicated in case of axillary lymph node involvement or advanced tumor stage ^[^^28^^]^. CMF was used in 27.0% and anthracycline-based in 64.9% of our patients, similar to the figures reported by Ben Dhiad et al. ^[^^27^^]^, who used adjuvant FAC in 50.0% of their patients.

Hormonal treatment is used due to strong positivity of hormone receptors; it was 85.7% in our studied patients (18 out of 21 cases) and 65% to 90% in the literatures ^[^^33^^-^^35^^]^. Most authors showed that the adjuvant hormonal therapy improved the survival rate but the efficacy was not obvious in our series, mostly related to use of tamoxifen in only 21 patients (18 patients with positive receptors and 3 with unknown receptor) ^[^^25^^, ^^35^^]^.

Local recurrence (LR) occurred in 4 cases (10.8% of all patients) after a median time of 23 months, compatible with results conducted by Bourhafour et al. ^[^^29^^]^ who found a LR of 8.5% after a median time of 36 months. Metastasis occurred in 11 cases (29.7% of all patients) after a median time of 22.5 months, but in the previous study metastasis occurred in 24.0% of patients after a median time of 12 months ^[^^29^^]^. This difference may be related to higher use of adjuvant chemotherapy in our study.

In our study, the 2-year OS and DFS were 81.6% and 68.4%, respectively, but they were 93% and 87.5% in a previous study conducted by Benchellal ^[^^30^^]^. The difference may be due to late-stage presentation of our patients. However, another study reported by Rachid et al, showed a lower 2-year DFS (50%) as more patients had advanced stage ^[^^9^^]^.

The 5-years OS varies among different series from 43% to 79% ^[^^27^^-^^30^^]^ *vs.* 60.5% for our population. The 5-year DFS was 52.6%, lower than that reported by Benchella et al. (75%) ^[^^30^^]^, and Park et al. (91%) ^[^^25^^]^. This difference may be due to late stage presentation of our patients.

Tumor size, positive lymph nodes, distant metastasis at the time of presentation of MBC and use of adjuvant chemotherapy are significant prognostic factors for DFS in our study and other studies ^[^^25^^, ^^27^^, ^^35^^]^, while none of the studied parameters was found as significant prognostic factors for OS. Other factors which have been reported by other investigators ^[^^27^^, ^^28^^, ^^30^^-^^35^^]^ such as age, grade, skin invasion, were insignificant in our study and this may be attributed to the small number of patients.

## Conclusion

MBC is a rare disease and has many similarities in clinical and histopathological characteristics to FBC. However, MBC is more often diagnosed at a more locally advanced stage. Surgical treatment remains the gold standard for MBC. Adjuvant therapy is based on retrospective studies of MBC conducted over the past 20 years using the guidelines for breast cancer in women. Future research for better understanding of this disease at national or international level is needed to improve the management and prognosis of MBC patients.
